# Prognosis of Cancer Patients with Severe Hyponatremia in the Emergency Department: A Retrospective Study from the National Cancer Center of China

**DOI:** 10.3390/curroncol32050245

**Published:** 2025-04-23

**Authors:** Qinglong Jiang, Xi Zhang, Chao Wang, Rong Qin, Rui Sun, Shengling Qin, Cong Zhao, Zhiyong Li, Wenjie Zhu, Minghua Cong

**Affiliations:** Department of Comprehensive Oncology, National Cancer Center/National Clinical Research Center for Cancer/Cancer Hospital, Chinese Academy of Medical Sciences and Peking Union Medical College, Beijing 100021, China; jqinglongsdu@126.com (Q.J.); xizhang1225@foxmail.com (X.Z.); wangpkuhsc@163.com (C.W.); rongrongqin@126.com (R.Q.); moonlet@126.com (R.S.); leslie_beloved@126.com (S.Q.); bipiao1982@163.com (C.Z.); 13341190168@163.com (Z.L.); studs@126.com (W.Z.)

**Keywords:** severe hyponatremia, oncologic emergency, hypoalbuminemia, mortality

## Abstract

Aim: The aim of this study was to analyze the clinical characteristics and prognostic factors of profound hyponatremia in solid cancer patients admitted to the oncologic emergency department. Methods: We gathered data retrospectively from cancer patients who visited the emergency department of the National Cancer Center of China between October 2019 and February 2023 with a serum sodium (Na) level of less than 125 mmol/L. The demographic and clinical characteristics, medical history, admission symptoms, laboratory parameters, and outcomes of the patients were recorded. Results: This study comprised 307 patients with severe hyponatremia in total. With 39.4% of all tumors being lung cancer (*n* = 121), nausea and vomiting were the most common admission symptoms for patients with severe hyponatremia. The 30-day mortality rate of profound hyponatremia cancer patients in the emergency department was 13.4%. The albumin level (*p* < 0.001), the hemoglobin level (*p* = 0.033), the TNM stage (*p* = 0.004), the Eastern Cooperative Oncology Group Performance Status (ECOG-PS) score (*p* < 0.001), hypocalcemia (*p* = 0.006), renal insufficiency (*p* = 0.035), and the efficacy of sodium supplementation (*p* = 0.006) were significantly associated with 30-day mortality. Binary logistic regression analysis showed that a lower albumin level (OR 0.924, 95% CI 0.861–0.991, *p* = 0.028) and higher ECOG score (OR 8.443, 95% CI 3.568–19.976, *p* < 0.001) were independent risk factors for 30-day mortality. The overall survival of emergency cancer patients with severe hyponatremia was also examined. The results of the COX regression analysis demonstrated that the efficacy of sodium supplementation (OR = 2.643, 95% CI 1.593–4.386, *p *< 0.001), a low albumin level (OR = 0.654, 95% CI 0.463–0.923, *p* = 0.016), the TNM stage (OR = 4.606, 95% CI 2.846–7.455), and a higher ECOG score (OR = 1.738, 95% CI 1.292–2.338, *p *< 0.001) were independent risk factors for overall survival. Conclusions: The clinical manifestations of severe hyponatremia in emergency cancer patients are varied. Hypoalbuminemia and a higher ECOG score are independent risk factors for 30-day mortality and overall survival. Severe hyponatremia patients with a high ECOG score and/or a low albumin level should be monitored and followed more closely.

## 1. Introduction

Hyponatremia is the most prevalent electrolyte imbalance encountered in clinical practice among cancer patients [1]. This condition is the primary expression of the Syndrome of Inappropriate Antidiuretic Hormone (SIADH), a frequently occurring condition in small-cell lung cancer. Clinically, it must be differentiated from hypovolemic hyponatremia brought on by significant gastrointestinal tract loss. When serum sodium (Na) falls below 125 mEq/L, it is referred to as severe hyponatremia [2]. Depending on the level of serum sodium concentration, the rate of development, and the patient’s prior general clinical status, the clinical signs of severe hyponatremia in cancer patients can range greatly, from asymptomatic to life-threatening [3]. Acute hyponatremia is linked to neurocognitive slowdown and migraines. Seizures and even death may result from severe hyponatremia.

Due to the annual growth of new cancer cases, the use of emergency services by cancer patients is also increasing [4,5]. Hyponatremia research is becoming more and more crucial since hyponatremia is one of the most frequent electrolyte imbalances witnessed in emergency departments. Hyponatremia has been found to be an independent adverse prognostic factor for cancer patients’ survival in earlier research [6,7,8]. A poorer prognosis has also been linked to hyponatremia that does not return to normal while a patient is receiving antitumor treatment [9]. There are also some data on cases presenting to general emergency departments with hyponatremia, and the risk factors and associated mortality related to hyponatremia have been studied and evaluated [10,11], but still, there are very limited data focusing on severe hyponatremia of oncologic emergency patients, especially in China.

The aim of this study was to assess the clinical features of severe hyponatremia and analyze the variables influencing mortality in patients admitted to the oncologic emergency department with profound hyponatremia.

## 2. Methods

### 2.1. Study Design and Data Collection

This retrospective study was conducted at the emergency department of the National Cancer Center of China and included patients diagnosed with severe hyponatremia between 1 October 2019 and 28 February 2023. Patient selection was based on histopathologically confirmed malignancy with a serum sodium level below 125 mEq/L on laboratory examination. Patients were excluded if they had received sodium supplementation before attending the emergency department. Patients with incomplete file information, those under the age of 18, and individuals admitted to the emergency department with cardiopulmonary arrest were excluded from this study. If the patient had more than one admission due to severe hyponatremia within the specified timeframe, the data from the first admission were assessed. Ethical approval for this study was granted by the Ethics Committee of the National Cancer Center/Cancer Hospital, Chinese Academy of Medical Sciences and Peking Union Medical College [No. 24/208-4488]. This study was conducted in accordance with the Declaration of Helsinki and good clinical practices.

Where available, the following variables were extracted from routinely performed measurements documented in the medical charts: baseline clinical characteristics, including patient demographics and comorbidities. Patients’ baseline vital signs and laboratory blood test results were also collected, especially for abnormalities in hemoglobin levels, albumin levels, and other electrolyte disturbances such as hypokalemia and hypocalcemia. Basic tumor data, including tumor site, pathological type, TNM stage, brain metastases, serosal effusion, and antitumor treatments, were also gathered for the patients in this study. This study strictly followed the inclusion and exclusion criteria, and two-person input ensured the accuracy of the data. The researchers extracted data from the hospital’s electronic medical record system and inputted it into a data collection list specifically constructed for this study. Every two researchers involved in data collection and management formed a team to supervise and verify the accuracy of data entry. Personal identification data were used to identify the patients during the collection process to protect the privacy of the patients. All patients treated in the National Cancer Center of China were followed up in the hospital follow-up system. We gathered the follow-up data until February 2024. The 30-day mortality and long-term survival were measured.

### 2.2. Statistical Analysis

We used SPSS version 27.0 (Chicago, IL, USA) in our analysis. Descriptive data were summarized using means, standard deviations, frequencies, and percentages. The Pearson Chi-square independence test (univariate approach) was used between study variables and 30-day mortality of the patients to determine if there was a statistically significant association between the two groups’ proportions. Variables significantly associated with 30-day mortality (*p* ≤ 0.05) were incorporated into the logistic regression model to determine the odds ratio (OR) of clinically significant data, and to explore the risk factors associated with the short-term survival outcome in patients with severe emergency hyponatremia. Survival analysis was performed utilizing the Kaplan–Meier method. Possible prognostic factors influencing survival were first evaluated by univariate analysis (log-rank test). Only parameters that showed significance in univariate analysis were further analyzed by multivariate analysis (Cox proportional hazards test, forward conditional method). Statistical significance was defined as a *p* value of less than 0.05.

## 3. Results

Among 78,575 patients who went to the emergency department of the National Cancer Center of China between 1 October 2019 and 28 February 2023, 307 patients (155 males and 142 females) with severe hyponatremia and pathologically confirmed solid tumors were included in this study. The mean age of the patients included in this study was 61.27 ± 10.43. The peripheral blood sodium index of all patients was lower than 125 mmol/L, and the mean value was 119.49 ± 4.87 mmol/L (90.3 mmol/L–124.9 mmol/L). Based on laboratory tests, 53 individuals were found to have hypocalcemia, and 49 patients were found to have hypokalemia. There were 232 patients with hypoproteinemia, with an average human blood albumin level of 35.04 ± 6.69 g/L. With an average HGB level of 115.86 ± 24.84 g/L, there were 146 individuals with varying degrees of anemia. There were 54 patients with renal insufficiency (Table 1). Of the 303 patients who underwent emergency sodium supplementation, 48 patients returned to normal blood sodium, and 161 patients did not seek emergency treatment after the blood sodium index was improved to a mild or moderate level of low sodium, while for 98 patients, blood sodium was not reviewed after sodium supplementation or blood sodium was still lower than 125 mmol/L.

**Figure 1:**
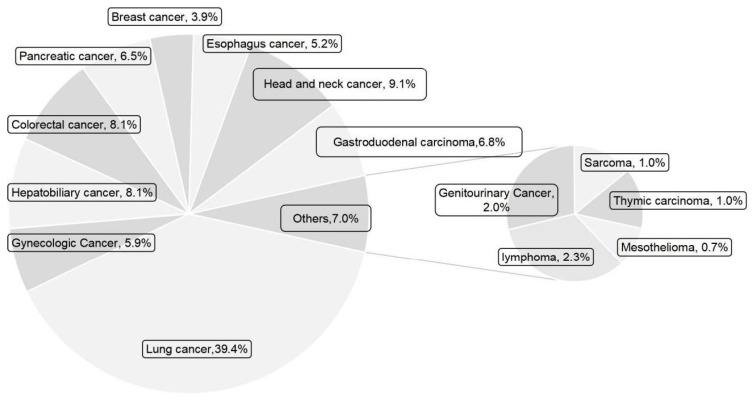
Classification of cancer types in emergency patients with severe hyponatremia.

**Figure 2:**
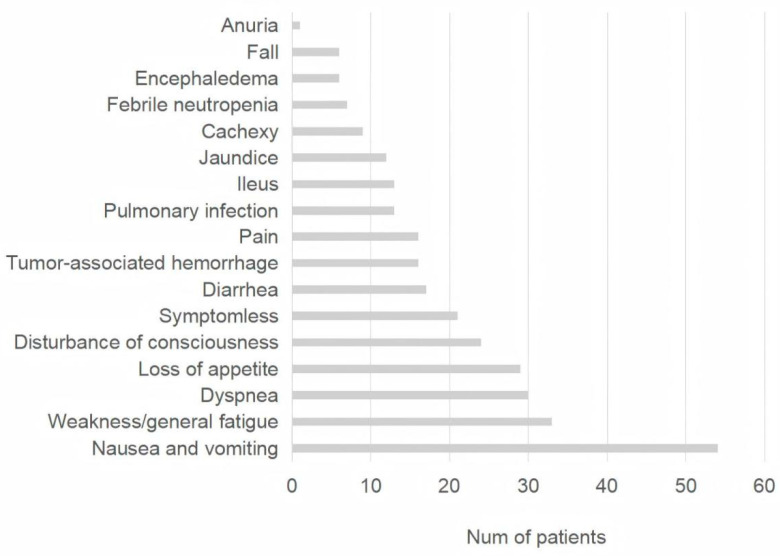
Chief complaints of cancer patients with severe hyponatremia in the oncologic emergency department.

The incidence of severe hyponatremia in emergency cases of various tumor types is shown in Figure 1. Lung cancer was the most frequent type of cancer, followed by head and neck cancer, colorectal cancer, hepatobiliary cancer, gastric cancer, esophageal cancer, etc. Of the 231 patients who were in clinical stage IV, 48 had brain metastases. In total, 183 patients received systemic chemotherapy, and 94 patients received immune checkpoint inhibitor therapy. Additionally, we calculated the main complaints raised by patients who came to the emergency room with tumors and severely low sodium. Vomiting was the most common symptom and complaint in 54 cases. This was followed by 33 cases of fatigue, 30 cases of dyspnea, 29 cases of inability to eat, and 24 cases of consciousness disturbance. There were 21 asymptomatic patients who came to the emergency room for sodium supplementation only because of laboratory results suggesting severe hyponatremia. Figure 2 shows the distribution of recorded symptoms.

When the 30-day mortality of severe hyponatremia patients in the oncologic emergency department was evaluated, the mortality rate was determined to be 13.4% (*n* = 41). Univariate analysis revealed that the factors impacting the 30-day mortality were serum albumin level (*p* < 0.001), hemoglobin level (*p* = 0.033), TNM stage (*p* = 0.004), the ECOG-PS score (*p* < 0.001), hypocalcemia (*p* = 0.006), renal insufficiency (*p* = 0.035), and the efficacy of sodium supplementation (*p* = 0.006) (Table 1). Binary logistic regression analysis showed that a lower serum albumin level (OR 0.924, 95% CI 0.861–0.991, *p* = 0.028) and a higher ECOG score (OR 8.443, 95% CI 3.568–19.976, *p *< 0.001) were independent risk factors for 30-day mortality (Table 2).

**Figure 3:**
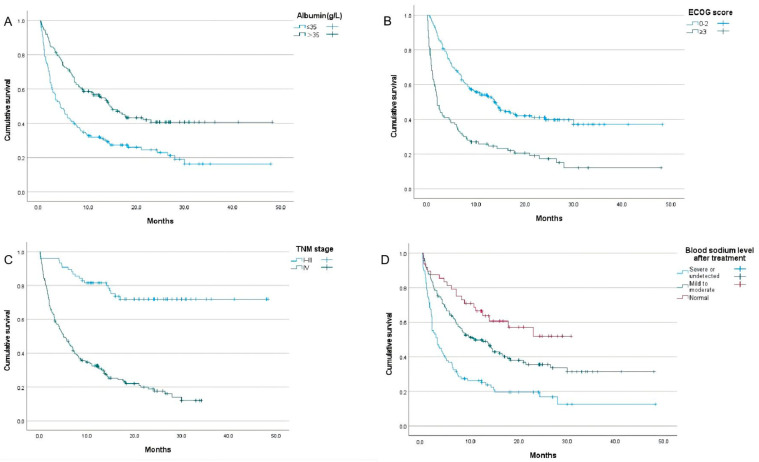
Survival analysis showed that hypoalbuminemia (**A**), a higher ECOG score (**B**), the TNM stage (**C**), and the efficacy of sodium supplementation (**D**) are the prognostic factors of overall survival.

The long-term results of patients with severe hyponatremia were investigated in this study. The survival analysis showed that a lower albumin level (*p* < 0.001), a lower hemoglobin level (*p* = 0.030), the TNM stage (*p* < 0.001), brain metastasis (*p* = 0.005), a higher ECOG score (*p* < 0.001), hypocalcemia (*p* = 0.020), renal insufficiency (*p* = 0.033), and the efficacy of sodium supplementation (*p* ≤ 0.001) were significantly associated with the overall survival of severe hyponatremia cancer patients in the oncologic emergency department (Table 3). The results of the COX regression analysis demonstrated that a lower albumin level (OR = 0.654, 95%CI 0.463–0.923, *p* = 0.016), the TNM stage (OR = 4.606, 95% CI 2.846–7.455, *p* < 0.001), a higher ECOG score (OR = 1.738, 95% CI 1.292–2.338, *p* < 0.001), and the efficacy of sodium supplementation (OR = 2.643, 95% CI 1.593–4.386, *p* < 0.001) were independent risk factors for overall survival (Table 3, Figure 3).

## 4. Discussion

Hyponatremia is a highly prevalent electrolyte imbalance in the emergency department and is associated with a high mortality rate, especially when severe hyponatremia occurs [12]. In addition, people with tumors may experience severe hyponatremia and need emergency care as a result of the disease or the antitumor therapy [5]. The following are some of the causes of cancer patients presenting to the emergency room with severe hyponatremia: firstly, reactions of the gastrointestinal tract to antitumor therapy, including nausea, vomiting, and diarrhea; secondly, tumor-induced gastrointestinal compression that interferes with eating, or brain edema that results in nausea and vomiting; thirdly, long-term insufficiencies in end-stage cancer patients manifesting systemic weakness, as patients with severe hyponatremia may experience neurological symptoms such as confusion, a changed level of consciousness, seizures, and focal neurological deficiency; lastly, patients may also exhibit asymptomatic hyponatremia [6,12], meaning that the low Na level was discovered accidentally and no hyponatremia-related symptoms were seen.

In this study, we analyzed the epidemiological characteristics, clinical manifestations, and risk factors influencing the short-term emergency outcome and long-term prognosis of cancer patients with severe hyponatremia in an oncologic emergency department. The greatest percentage of those patients had lung cancer, which was followed by malignancies of the head and neck and digestive tract. According to cancer data from China, lung cancer is the malignant tumor with the highest incidence [4]. With advancements in targeted therapy and immunotherapy, an increasing number of lung cancer patients are achieving long-term survival. The most frequent cancer linked to hyponatremia is small-cell lung cancer (SCLC), which usually results from SIAD and impacts about 25% of patients diagnosed with SCLC [5]. For patients with head and neck tumors and digestive system tumors, severe hyponatremia is usually caused by eating disorders, insufficient intake, or digestive reactions caused by antitumor therapy. This study did not find an association between patients receiving immunotherapy or chemotherapy and severe hyponatremia in emergency cancer patients.

Patients with severe hyponatremia may exhibit a variety of symptoms depending on the depth of hyponatremia and the duration of occurrence [13]. Acute hyponatremia is associated with headaches and neurocognitive slowing. It was reported that serious neurological findings such as confusion, coma, and convulsions were more common when the serum sodium concentration was below 120 mmol/L in the emergency department; however, there was no statistically significant difference between hyponatremia severity groups [14]. Another study showed that most patients presented with symptoms unrelated to hyponatremia [15]. The epidemiology and characteristics of hyponatremia in cancer emergency patients would be different from those in general hospital emergency patients. In our study, when the complaints of cancer patients who were considered to have severe hyponatremia were examined, it was observed that the patients most frequently presented with complaints of vomiting, followed by fatigue, dyspnea, oral intake disorder, and consciousness disturbance.

The reported mortality rates associated with severe hyponatremia vary widely, from 6.5% to 28.9% [14,16,17]. There are few data on mortality from severe hyponatremia in emergency cancer patients. In our study, the 30-day mortality rate of profound hyponatremia of cancer patients in the emergency department was 13.4%. The results of the survival analysis showed that the serum albumin level and ECOG score of cancer patients significantly affected the emergency mortality of patients with severe hyponatremia. Serum albumin has many important physiological functions in the body. Hypoalbuminemia can induce fluid extravasation, leading to intravascular volume depletion and increased ADH stimulation, resulting in hyponatremia [18]. Previous studies have shown that low albumin levels are associated with increased mortality in critical illnesses [19,20]. Researchers found that for patients with hyponatremia, hypoalbuminemia was identified as an independent predictor of mortality in multivariate analysis [21]. Performance status is an important indicator of general well-being and the ability to perform activities of daily living in patients with cancer. Performance status has also been repeatedly shown to predict important clinical outcomes, including quality of life, chemotherapy toxicity, response to chemotherapy, terminal illness, progression-free survival, and overall survival in patients with cancer [22,23].

Prior research has indicated that the survival of cancer patients may be impacted by hypoproteinemia and performance status [21,22]. The 30-day emergency mortality and long-term survival of emergency cancer patients with severe hyponatremia are negatively impacted by lower albumin levels and poor performance status, which indicate worse resistance and more severe disease. While actively treating hyponatremia, physicians may find that increasing patients’ performance status and administering albumin supplements can improve their prognosis.

The strength of our study is that this is the first detailed, comprehensive study examining the risk factors of mortality in cancer patients admitted to the emergency department with severe hyponatremia. Our study involved more than 300 patients with a wide variety of solid malignancies, as well as diverse demographic features, cancer stages, and treatment experiences. There are also some limitations in this study. First, the present work was a retrospective and single-center study with a limited number of patients, which may limit its generalizability to other settings. Second, we were unable to assess the patients’ volume status. We also do not have data on urine osmolality and spot urine Na for all patients. Third, the present study was conducted during the Coronavirus Disease 2019 (COVID-19) pandemic, which may have led to selection bias. We did not follow up on the cause of their deaths for all the patients. The inclusion of all-cause mortality is another limitation. Further prospective analysis of a larger population is needed to confirm our findings that the ECOG score and serum albumin parameters are prognosis markers in cancer patients with severe hyponatremia presenting to the emergency department.

## 5. Conclusions

Severe hyponatremia is a common emergency in tumor patients. The clinical manifestations of severe hyponatremia in emergency cancer patients are varied. Vomiting is the chief admission symptom. The 30-day mortality rate of profound hyponatremia cancer patients in the emergency department was 13.4%. A lower albumin level and a higher ECOG score are independent risk factors for both 30-day mortality and overall survival of emergency cancer patients with severe hyponatremia. In addition, the TNM stage and the effect of emergency sodium supplementation also significantly affect the prognosis of tumor patients with severe hyponatremia in the emergency department, suggesting that we need active and standardized sodium supplementation in emergency treatment. Physicians should be aware that severe hyponatremia causes death in emergency cancer patients and that mortality is more common in patients with these risk factors; accordingly, severe hyponatremia patients with a high ECOG score and/or a low albumin level should be monitored and followed more closely.

## Figures and Tables

**Table 1:** Clinical characteristics of cancer patients with severe hyponatremia in the emergency department.

Variables	Total (*n* = 307)	Death (*n* = 41)	Alive (*n* = 266)	*p* Value
Age (years)	61.27 ± 10.43	62.63 ± 11.69	61.06 ± 10.22	0.370
Gender				0.740
male	165	21	144	
female	142	20	122	
Serum sodium (mmol/L)	119.49 ± 4.87	119.58 ± 6.20	119.48 ± 4.65	0.901
Serum albumin (g/L)	35.04 ± 6.69	30.42 ± 6.19	35.75 ± 6.49	<0.01
HGB (g/L)	115.86 ± 24.84	108.15 ± 32.83	117.05 ± 23.23	0.033
Chemotherapy history				0.866
Yes	183	24	159	
No	124	17	107	
Immunotherapy history				0.467
Yes	94	10	84	
No	213	31	182	
Tumor type				0.052
Small-cell cancer	56	3	53	
Others	251	38	213	
TNM stage				0.004
I–III	76	3	73	
IV	231	38	193	
Brain metastasis				0.489
Yes	48	8	40	
No	259	33	226	
ECOG score				<0.01
0–2	207	8	199	
≥3	100	33	67	
Hyponatremia history				0.668
Yes	56	9	47	
No	238	32	206	
Hypocalcemia				0.006
Yes	53	14	39	
No	254	27	227	
Hypokalemia				0.803
Yes	49	6	43	
No	258	35	223	
Blood sodium after treatment				
Normal	48	4	44	0.006
Mild to moderate	161	15	146	
Severe or undetected	98	22	76	
Renal insufficiency				0.035
Yes	54	12	42	
No	253	29	224	

**Table 2:** Binary logistic regression analysis of 30-day mortality of severe hyponatremia in the oncologic emergency department.

Parameters	B	SE	Wald	Df	Sig	Exp(B)	95% CI
Serum albumin	−0.079	0.036	4.859	1	0.028	0.924	0.861–0.991
HGB	0.004	0.007	0.329	1	0.566	1.004	0.990–1.019
TNM stage	0.892	0.666	1.794	1	0.180	2.439	0.661–8.992
ECOG score	2.133	0.439	23.569	1	0.000	8.443	3.568–19.976
Renal insufficiency	0.111	0.460	0.058	1	0.809	1.118	0.454–2.754
Hypocalcemia	0.376	0.458	0.676	1	0.411	1.457	0.594–3.571
Efficacy of sodium supplementation	0.369	0.659	0.314	1	0.575	1.446	0.398–5.262

**Table 3:** Analysis of the overall survival in cancer patients with severe hyponatremia presenting to the emergency department.

Variables	Num.	Univariate Analysis *p* Value	Multivariate Analysis *p* Value	OR (95% CI)	95% CI Lower	95% CI Upper
Age (years)		0.777				
≤60	137					
>60	270					
Gender		0.587				
male	165					
female	142					
Albumin (g/L)		<0.001	0.016	0.654	0.463	0.923
≤35	146					
>35	161					
HGB (g/L)		0.030	0.621	1.081	0.793	1.475
≤115	149					
>115	158					
Chemotherapy history		0.163				
Yes	183					
No	124					
Immunotherapy history		0.340				
Yes	94					
No	213					
Tumor type		0.212				
Small-cell cancer	56					
Others	251					
TNM stage		<0.001	<0.001	4.606	2.846	7.455
I–III	76					
IV	231					
Brain metastasis		0.005	0.082	1.399	0.958	2.043
Yes	48					
No	259					
ECOG score		<0.001	<0.001	1.738	1.292	2.338
0–2	207					
≥3	100					
Hyponatremia history		0.097				
Yes	56					
No	238					
Hypocalcemia		0.020	0.783	0.948	0.646	1.391
Yes	53					
No	254					
Hypokalemia		0.296				
Yes	49					
No	258					
Blood sodium after treatment		<0.001	<0.001	2.643	1.593	4.386
Normal	48					
Mild to moderate	161					
Severe or undetected	98					
Renal insufficiency		0.033	0.434	1.155	0.805	1.657
Yes	54					
No	253					

## Data Availability

The data that support the findings of this study are not publicly available due to them containing information that could compromise the privacy of research participants but are available from the corresponding author Minghua Cong (e-mail: congmh@cicams.ac.cn) upon reasonable request.
